# A comprehensive review on emerging trends in the dynamic evolution of digital addiction and depression

**DOI:** 10.3389/fpsyg.2023.1126815

**Published:** 2023-02-08

**Authors:** Turgut Karakose, Bilal Yıldırım, Tijen Tülübaş, Abdurrahman Kardas

**Affiliations:** ^1^Department of Education, Kutahya Dumlupınar University, Kutahya, Türkiye; ^2^Department of Education, Istanbul Sabahattin Zaim University, Istanbul, Türkiye; ^3^District Director of National Education, Ministry of National Education, Siirt, Türkiye

**Keywords:** digital addiction, depression, science mapping, bibliometrics, problematic use

## Abstract

**Introduction:**

Using digital addiction as an umbrella term to cover any type of addictions to digital technologies such as the internet, smartphones, social media, or video games, the current study aimed to reveal the intellectual structure and evolution of research addressing digital addiction-depression relationship.

**Methods:**

The study combined bibliometric and science mapping analysis methods for this purpose. Data for the study was gathered from Web of Science Core Collection after a comprehensive process of data search/extraction, and 241 articles were included in the final data set. A period-based, comparative science mapping analysis was performed using the SciMAT software.

**Results:**

The analysis of data over three periods, Period 1 (1983-2016), Period 2 (2017-2019), and Period 3 (2020-2022) showed that internet addiction was the most significant theme across all three periods, which was followed by social media addiction. Depression, which emerged as a significant theme during Period 1, was later covered under anxiety disorder theme. Research interest was mostly on factors related to both addiction and depression such as cognitive distortion, insomnia, loneliness, self-esteem, social support, alexithymia, as well as cybervictimization or academic performance.

**Discussion:**

The results suggested that much research is warranted on the digital addiction-depression relationship in different age cohorts, especially children and elderly. Similarly, the current analysis showed that this line of research particularly focused on internet, gaming and social media addiction, and evidence with regard to other types of digital addiction or related compulsive behaviors was almost absent. In addition, research was mostly inclined to understanding cause-effect relationships, which is significant, but preventive strategies seemed to be barely addressed. Likewise, the smartphone addiction-depression relationship arguably garnered less research interest, so future research would contribute to the field in this respect.

## 1. Introduction

In the current era of digital revolution, digital technologies have become integral to many aspects of daily life, and transformed the functioning of societies across the globe (Zhou et al., 2022). With the advent of mobile technologies and complex digital systems such as artificial intelligence or the Internet of Things, many digital devices and systems such as laptops, smartphones, tablets, social networking sites, or other online platforms have become prominently used among people of all ages (Bucci et al., 2019; Wiraniskala and Sujarwoto, 2020; Chang et al., 2022). Today, it is considered that nearly half of worlds’ population has already gone digital, and digital interactions are increasingly urging people, even those with limited digital resources, to change their lifestyles (Elliot, 2018).

Digital technologies have contributed to people’s quality of life from several aspects as they are used for a variety of reasons ranging from exploration, information sharing, and self-documentation, and facilitating learning to entertainment, communication, relationship building/maintenance, and socialization (Chakraborty, 2016; Hughes and Burke, 2018; Foroughi et al., 2022). Despite these benefits, over-exposure to these digital technologies or their problematic use have raised serious concerns with regard to their adverse effects on peoples’ physical and mental health, particularly through causing digital addiction (Bucci et al., 2019; Ho, 2021; Peng et al., 2022).

In the literature, a variety of concepts are used to address problematic or addictive use of digital technologies, usually referring to one type of these technologies such as the problematic, excessive, compulsive, or pathological use of the internet, as well as the smartphone, social media or internet/smartphone/social media addiction. Likewise, several compulsive behaviors are investigated in relation to these specified types of addictions such as cyberchondria, cyberstalking, cybervictimization, digital hoarding, and compulsive online shopping. Some scholars use the term ‘the problematic use of the internet (PUI) to encompass all these potentially problematic internet-related behaviors, and abstain from using the term “addiction” because a consensus over its diagnosis as an addiction has not been reached yet (Fineberg et al., 2022; Moretta et al., 2022). On the other hand, the term “digital addiction” is used as an overarching term that encompasses any type of problematic interaction with digital technologies, be it online or offline (Christakis, 2019; Meng et al., 2022). Digital addiction is then used to refer to dependence on using digital devices or technologies in a way that leads to behavioral symptoms similar to any addictive disorder (Joseph and Hamiliton-Ekeke, 2016).

In the current study, we preferred to use the term “digital addiction” as an umbrella term to refer to any type of behavioral addiction resulting from human-machine interaction, and characterized with excessive-impulsive use, withdrawal, tolerance, adverse effects, craving, and/or affect dysregulation (Griffiths, 1996; Cerniglia et al., 2017; Basel et al., 2020; Ibrahim et al., 2022). In that vein, digital addiction involves several sub-types such as addiction to the internet, social media, smartphones, online/video games, or cyber-relationships (Alrobai et al., 2019; Meng et al., 2022), and digital addicts exhibit the previously-defined six core criteria of addiction, i.e., salience, mood modification, tolerance, withdrawal symptoms, conflict, and relapse (Al Mamun and Griffiths, 2019).

Like in other forms of addiction, scholars contend that addiction to digital technologies fundamentally results from the dysfunction of brain reward circuits, and the positive or negative reinforcement of environmental stimuli plays a significant role in its development (Weinstein and Lejoyeux, 2015; Nam et al., 2018). Research into the brain activities of digital addicts such as internet or video game addicts evidence alterations in their brain activity which are commonly associated with both chemical (i.e., drug addiction) or non-chemical (i.e., behavioral) addictions, and underlies that addiction develops as a result of a deficient reward system (Kuss and Griffiths, 2012; Weinstein and Lejoyeux, 2015; Chun et al., 2017). The intermittent rewards provided by digital contents (e.g., social media services, texts, videos, push notifications) are considered to activate reward circuits in the brain, and lead to dependency formation (Peper and Harvey, 2018). Prolonged addiction could even damage the function of the brain circuitry regulating these reward and reinforcement mechanisms, which in turn impact emotion regulation systems negatively (Wise and Morales, 2010; Polter and Kauer, 2014).

Digital addiction as a newer form of behavioral addiction has attracted increasing research interest across the globe (Karakose et al., 2022b), and it is revealed to harm peoples’ well-being both directly or indirectly through causing emotional impairments such as depression (Carli et al., 2013; Demirci, 2019; Guo et al., 2020; Ibrahim et al., 2022; Yang et al., 2022). Depression, which is distinguished from the usual mood fluctuations or short-lived emotional responses of people (World Health Organization (WHO), 2022), is considered to be a serious, prolonged health condition that manifests itself as sadness, loss of interest, sleep problems, fatigue, changes in appetite, purposeless activity, feelings of guilt or worthlessness, difficulty thinking, poor concentration, and decreased life satisfaction (Koo, 2018). When not treated, depression entails public health hazard through causing significant chronic diseases and even suicides as well as damaging psychological and social functioning of individuals (Wang et al., 2018; Yoon et al., 2019). Depression is even considered to be a leading cause of disability worldwide (World Health Organization (WHO), 2022).

Considering the potential harmful outcomes of both digital addiction and depression, researchers have so far paid significant attention to the relationship between digital addiction and depression. Indeed, several studies revealed strong associations between depression and internet addiction (Demir and Kutlu, 2016; Hoare et al., 2017; Lee et al., 2019; Marzilli et al., 2020), smartphone addiction (Sohn et al., 2019; Chang et al., 2022; Peng et al., 2022), social media addiction (Donnelly and Kuss, 2016; Al Mamun and Griffiths, 2019; Ho, 2021; Foroughi et al., 2022), and video game addiction (Krossbakken et al., 2018; Cudo et al., 2022; Hu et al., 2022; Zhou et al., 2022).

Although research addressing the associations between digital addiction and depression have revealed significant correlations between the two, the results continue to display an egg-chicken situation with regard to casual relationships between digital addiction and depression (Geng et al., 2021). One line of research shows that digital addiction could predict depression (Bian and Leung, 2015; Rozgonjuk et al., 2018; Wartberg et al., 2019) while another line of research suggests the opposite, indicating that depression could predict digital addiction (Guillot et al., 2016; Zhou et al., 2017; Kim and Koh, 2018; Yuan et al., 2021). There is also another line of research that addresses correlations between the two, and reveals a reciprocal relationship between digital addiction and depression (Chen et al., 2020; Stankovi et al., 2021; Morita et al., 2022).

Yang et al. (2022) briefly summarizes the theoretical perspectives underlying these three courses of research. Accordingly, research adhering to secondary psychiatric disorder hypothesis predicate on the assumption that psychiatric disorders precede the development of addictive behavior, and thus depression could lead some people to engage in excessive use of digital technologies to compensate this negative emotional state. On the other hand, research adhering to the secondary addictive disorder hypothesis dwells on the assumption that addiction could lead to psychological disorders through causing deficit reward-seeking, behavioral control, and decision-making mechanisms. The third line of research proposes bidirectional relationships between addiction and depression, indicating that this reciprocal relationship between the two could increase vulnerability to both. When the results of all three courses of research considered collectively, it can be asserted that the association between depression and digital addiction could be unidirectional or bidirectional (Yang et al., 2022).

The association between digital addiction and depression is also explained through some other hypothesis such as social displacement and mood enhancement hypothesis (Liang et al., 2016; Fineberg et al., 2022), and cognitive-behavioral model of pathological Internet use (Peng et al., 2022). Social displacement hypothesis proposes that social interactions *via* digital technologies decrease the time spent for face-to-face interactions with family or friends, and as a result, people have difficulty maintaining their social resources. Eventually, engaging in online/remote interactions could not compensate people’s need to maintain intimate relationships and causes feelings of alienation or loneliness rather than security and belonging. According to the hypothesis, these negative feelings are most likely to result in depression (Kraut et al., 1998; Hall et al., 2019; Vujić and Szabo, 2022). On the other hand, research also shows that people, particularly adolescents and young adults with depression tend to avoid real-life communication as they feel frustrated and intimidated. As a result, they might be more likely to spend excessive time on digital platforms and develop digital addiction (Zhang et al., 2014; Özbek and Karas, 2022).

The mood enhancement hypothesis claims that people with negative emotional states like depression tend to seek entertainment in a virtual world, and their prolonged exposure to these digital platforms is likely to cause addiction. As a result, the hypothesis suggests that the higher the level of depression, the more serious the digital addiction (Jeong et al., 2019; Yi and Li, 2021). Cognitive-behavioral model of pathological internet use hypothesis, on the other hand, suggests that people with depression are more likely to develop addiction as they are more easily enthralled by the internet. The theory was first developed to understand internet addiction (Davis, 2001) but recent research on other digital addictions such as smartphone or gaming addiction refer to the theory as they all share features (Billieux et al., 2015; Peng et al., 2022). As Lopez-Fernandez (2018) later explains, the theory distinguishes two pathological uses of the internet (i.e., specific activities like online gaming and general excessive use including global set of online behaviors), and proposes that specific internet use is more related to maladaptive cognitions while the general use could result from several external factors such as social isolation, lack of purpose, or procrastination.

Research addressing the digital addiction-depression relationship also underlines that adolescents and young adults are the most vulnerable groups to both depression and digital addiction because they often have low self-control, and tend to behave more impulsively (Liang et al., 2016; Jozefiak et al., 2019; Fineberg et al., 2022; Ibrahim et al., 2022). Likewise, adolescents are considered to experience more negative emotions and depression, especially in the case of family-related problems (Sela et al., 2020). There is also growing evidence that adolescents or young people are more prone to digital addiction as they spent more time using digital technologies (Lin et al., 2016; Al Mamun and Griffiths, 2019; Yoon et al., 2019; Haand and Shuwang, 2020; Arikan et al., 2022).

### 1.1. The purpose of the current study

The growing evidence on digital addiction in general, and on its association with depression in particular have not only raised awareness but also prompted concerns over their significant negative impacts on the current and future generations. Despite being a rather newer type of behavioral addiction, the literature on digital addiction and its sub-types such as internet, smartphone, social media, or video game addiction have accumulated a significant knowledge base, and offered insights on its association with depression. In addition to many empirical and cross-sectional studies, scholars also contributed to this field through several reviews addressing the relationships between depression and a particular sub-type of digital addiction (e.g., Carli et al., 2013; Baker and Algorta, 2016; Seabrook et al., 2016; Frost and Rickwood, 2017; Elhai et al., 2017a, b; Sohn et al., 2019; Yoon et al., 2019). Although these traditional reviews were significant in terms of providing cumulative results with regard to addiction-depression relationship across several contexts, to our knowledge, the literature still lacks a comprehensive study that delineates the conceptual and intellectual growth of this research field, which could particularly guide future studies through highlighting the well and under-investigated aspects as well as delineating the prominent themes, scholars and publications in the field. Hence, the present study was conducted to address this gap in the literature and aimed to reveal the conceptual and intellectual evolution of research focusing on the relationship between digital addiction and depression through the bibliometric and science mapping analysis of this research field. The study particularly seeks answers to the following questions:

- What is the volume and growth trajectory of research on digital addiction and depression?
- What are the leading journals, scholars, articles, and countries in the digital addiction and depression research field?
- What themes have guided digital addiction and depression research field during its evolution?
- What relationships have been observed between digital addiction and depression across different periods of its evolution?
- What are the emergent future directions in the digital addiction and depression research field?

## 2. Materials and methods

### 2.1. Study design

The study uses meta-data to analyze the digital addiction-depression research field, and combines the bibliometric and science mapping analysis to seek answers to the main research questions. Their combination enables to identify the conceptual structure, intellectual evolution, and bibliometric performance of the digital addiction-depression knowledge domain (Cobo et al., 2011a; MacFadden et al., 2021).

### 2.2. Data search and identification

In bibliometric and science mapping studies, digital databases such as Web of Science (WoS), Scopus or PubMed are often used to search and identify data. In the current study, we preferred to use WoS Core Collection to collect data because WoS has a broader coverage of high-quality scientific journals, and is one of the world’s premier sources of research data in many disciplines. Therefore, using WoS reduces the risk of articles being missed and thereby helps to prevent data loss (Mongeon and Paul-Hus, 2016). WoS is also cited to be the optimum database for conducting bibliometric analysis because it provides reliable and detailed information about the publications (Martínez et al., 2015).

**Figure 1:**
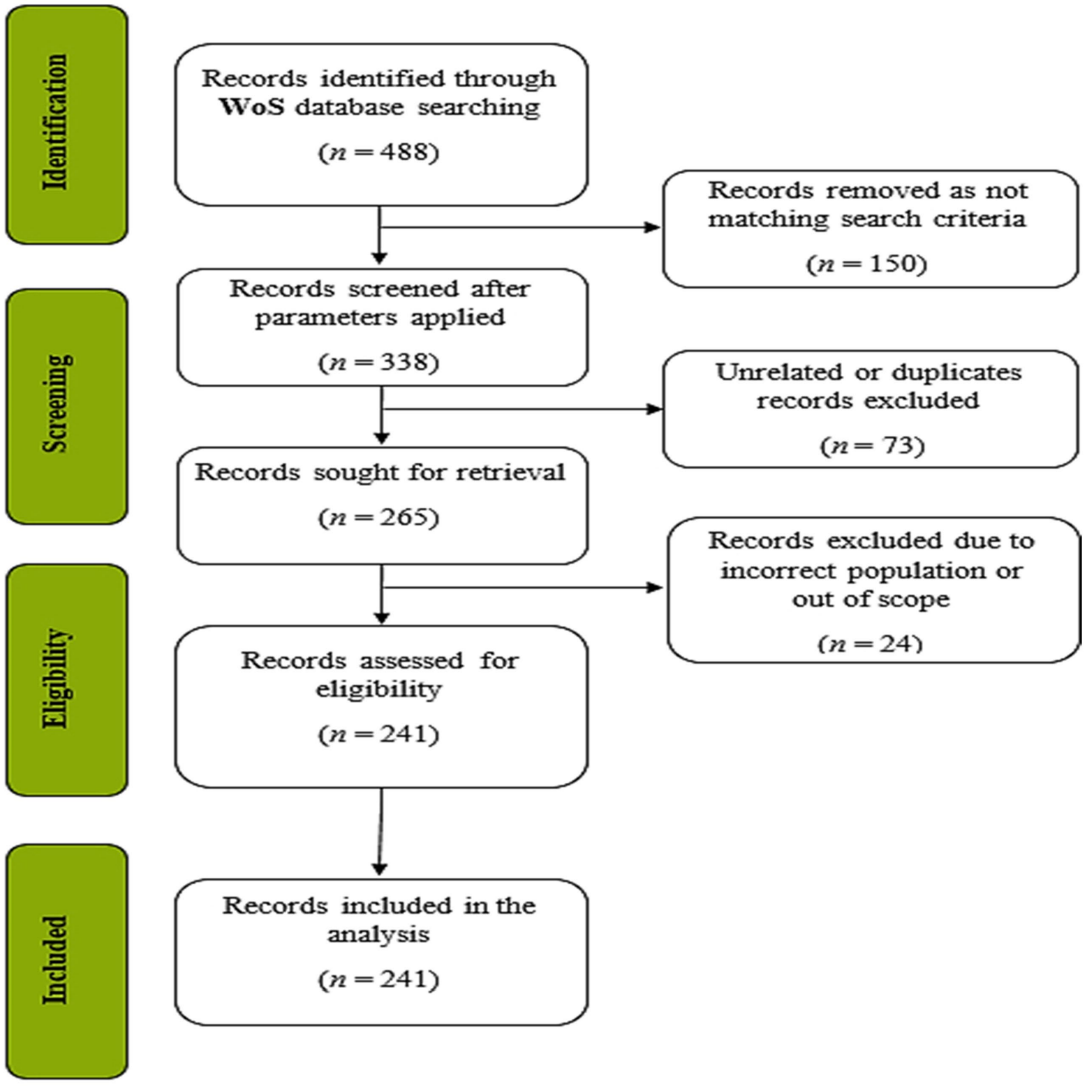
PRISMA flow diagram.

During data search and extraction, we followed the three-step procedure of searching/defining data, extracting/cleaning data, and, analyzing the data (Hallinger and Kulophas, 2019), and reported the search/selection process according to the PRISMA (Preferred Reporting Items for Systematic Reviews and Meta-Reviews) guidance (Moher et al., 2009; see Figure 1).

Data selection was made according to the inclusion/exclusion criteria in Table 1. Accordingly, we included journal articles written in English, and indexed in WoS while excluding books, book chapters, conference proceedings, and dissertations as well as articles written in other languages or indexed in other databases.

**Table 1:** Inclusion/exclusion criteria.

Criteria	Included	Excluded	Rationale
*Language*	English	Other languages	English is internationally used as the language of science Single language helps yield comprehensive conceptual analysis
*Document type*	Journal articles	Books, book chapters, dissertations, conference proceedings	We targeted peer-reviewed, high-quality publications
*Database*	WoS	Other databases (e.g., Scopus, PubMed, Google Scholar)	WoS covers wide scope of research from all fields of study

The following keyword string was used to perform a keyword search on the WoS database on 27 August 2022:

*TS=(“digital addiction” OR “internet addiction” OR “social media addiction” OR “smartphone addiction” OR “mobile phone addiction” OR “digital media addiction” OR “virtual addiction” OR “technology addiction” OR “computer game addiction” OR “gaming addiction” OR “mobile addiction” OR “digital game” OR “virtual game” OR “online game” OR “social network” OR “online shopping addiction” OR “cybersex addiction” OR “online movies” OR “social media” OR “gadget addiction” OR “mobile apps addiction” OR “internet gaming” OR "gaming disorder" OR “sms addiction” OR “mobile media” OR “problematic use” OR “problematic smartphone use” OR “problematic social media use” OR “game addiction” OR “digital leisure” OR “selfie addiction”) AND TS=(“depression”)*

While selecting the keywords, we first conducted a detailed literature review on digital addiction, and its sub-types. We prepared a list of keywords based on this initial review, and later we consulted two field experts before we formed the final list. Using the keyword string above, we conducted the first search on WoS. This initial search returned 488 documents. Screening through this raw data, we identified that 150 documents did not match either one or more search criteria given in Table 1. After excluding these 150 documents, 338 documents remained. Skimming through the titles and abstracts of these 338 articles, we first identified that 73 of them were either duplicate, or were not addressing the relationship between addiction and depression, and excluded them from the data set. Later, remaining resources were checked for eligibility, and 24 more documents were excluded as they were evaluated to be out of scope or to provide insufficient information about its population. At this stage, we conducted a peer-debriefing to discuss over the inclusion/exclusion process, and reached a 95% consensus over the 241 documents selected for analysis.

### 2.3. Data extraction and analysis

For data analysis, we used SciMAT (Science Mapping Analysis Tool) software-version 1.1.04. First, we transferred the bibliometric data of 241 articles on SciMAT. Next, we combined keywords with similar meanings manually to increase the effectiveness of thematic analysis (Cobo et al., 2012; López-Robles et al., 2021). For instance, we combined “disorder” and “disorders,” or “behaviour” and “behavior.” Finally, we continued with overall bibliometric analysis, and science mapping analysis, respectively. Through bibliometric performance analysis, we identified the distribution of articles addressing digital addiction-depression relationship by their year of publication, the accumulated number of articles, and the average citations received per article (Zupic and Čater, 2015). Then, we conducted science mapping analysis using the SciMAT software tool (Cobo et al., 2012), and determined the conceptual structure and thematic evolution of the digital addiction-depression research. SciMAT allows for a combined analysis of scientific mapping and bibliometric performance, and enables to visualize and define topics/themes specific to a research field. More significantly, SciMAT enables to exhibit the thematic evolution of the research field over sequential time periods, and allows for comparative interpretations over its intellectual and conceptual development (Cobo et al., 2012; Batagelj and Cerinšek, 2013; Martínez et al., 2015; Chen, 2017).

The conceptual science mapping analysis was conducted following two steps: (i) *identification of research topics*, and (ii) *visualization of research themes and thematic networks* (Coulter et al., 1998; Cobo et al., 2011a, 2012; Martínez et al., 2015). During the first step, we created a standardized network of common words for each term using the keywords extracted from the dataset, and later we applied a clustering algorithm based on the co-occurrence of the keywords. Next, we identified research topics through applying the clustering algorithm to a normalized network of co-occurring words. The collection of closely related keywords formed a set of themes. Thus, the conceptual architecture with main and sub-themes were identified identify and visualized, which also delineated the thematic evolution of the research field. Following this initial step, we continued with the visualization of research themes and thematic networks. At this stage, we used two different tools to determine the themes related to digital addiction-depression research field: the strategic diagram and the thematic network. We first presented the themes in a four-quadrant, two-dimensional strategic diagram based on values of centrality (x-axis) and density (y-axis), Centrality values demonstrate the extent to which a cluster interacts with other clusters as well as the strength of their relationship. It is formulated as c = 10^x^Σekh. Intensity values, on the other hand, demonstrate the internal strength of the relationship between the keywords within a theme. It is formulated as d = 100 (Σeij/w).

**Figure 2:**
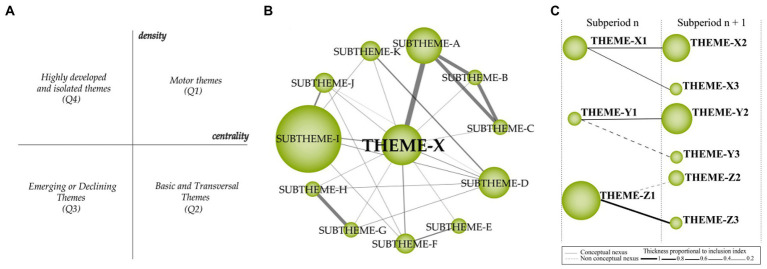
**(A)** Strategic diagram, **(B)** thematic network structure, and **(C)** thematic evolution structure (Cobo et al., 2012).

A conceptual analysis was performed based on co-word and h-index analyses, and divided research themes into four categories. Both results are presented within the biaxial strategic diagram (see Figure 2 for an example). The four categories in the strategic diagram are labeled clockwise as (i) motor themes (Q1), (ii) basic and transversal themes (Q2), (iii) emerging and declining themes (Q3), and (iv) highly-developed and isolated themes (Q1). To explain briefly;

- Motor themes quartile comprise themes with high centrality and density values. They are highly-developed, and the most significant themes for the development and structuring of the research field.
- Basic and transversal themes quartile comprise themes with high-centrality and low-density values. These themes are relevant to the field, but not well-developed due to lack of appropriate density, but have the potential to evolve into motor themes in the future due to their high centrality.
- Emerging or declining themes quartile includes themes with low centrality and density values. These themes are weakly-developed, or they represent topics marginal to the field, or topics that lost research interest in the meantime.
- Highly-developed and isolated themes quartile include themes with low centrality and high-density values. These themes are highly-developed but also highly specialized or peripheral for the field under investigation. They could also represent topics that lack appropriate background for the field.

Figure 2B shows an example of *thematic network structure,* which shows how strategic themes emerge in combination with other subthemes related to the field. The name of the most important (most central) keyword in the associated theme is used to label each thematic network. The keywords are interconnected, and the volume of the spheres align with the number of documents corresponding to each keyword. Similarly, the size of each circle aligns with the corresponding number of articles, while the thickness of the lines demonstrates the strength of the relationship between keywords.

Figure 2C shows an example of *thematic evolution map,* which is used to explore the time period, origin, and the evolution of the interrelationships of themes. The thematic evolution map includes a set of themes that emerged over consecutive time periods. The interrelationships between themes determine whether a theme belongs to a different thematic area or is a continuation of any one theme. Solid lines on the thematic map show where the same keywords are shared between themes, and dashed lines show common words being shared along with theme names. The thickness of the lines demonstrates the degree of the relationships, and the size of the circles align with the number of articles.

We also explored the conceptual links between themes from different periods using the inclusion index. The equation for the inclusion index is Ii = #(U∩V)/min(#U, #V) (Börner et al., 2003; Sternitzke and Bergmann, 2009). We created the thematic evolution map by combining the U theme with the V theme through conceptual linking (i.e., common keywords). A thematic connection between the U themes and the V themes exhibits commonalities and therefore their evolution. As the number of keywords shared by clusters across different periods increases, the conceptual evolution becomes more evident.

As mentioned earlier, SciMAT allows for analysis over consecutive periods to determine the thematic evolution of the research field under investigation. This period-based analysis is particularly suggested to save the data from uniformity (López-Robles et al., 2021). As the pioneering researchers in SciMAT analysis, Cobo and his colleagues suggest that periods could be determined based on three important issues: (1) events that caused key changes in the field, (2) including a sufficient number of articles in each period of analysis, (3) having an almost equitable number of articles in each period. They also underline that for a rigorous analysis, having an almost equitable proportion of articles for each period is more significant than having the same/similar range of years (Cobo et al., 2011a, 2011b; López-Robles et al., 2019). Taking these specifications into consideration, we divided the raw data into three periods depending on the volume of the relevant literature (i.e., the number of publications): Period 1 (1983–2016), Period 2 (2017–2019), and Period 3 (2020–2022).

## 3. Results

### 3.1. Overall bibliometric analysis

We performed a bibliometric performance analysis to determine the accumulated number of publications, the distribution of articles according to publication year, citations per article, most influential authors, most influential journals, most cited articles, and most productive countries with regard to research addressing digital addiction-depression relationship. Thus, we were able to demonstrated the international impact of publications in digital addiction-depression research field (Zupic and Čater, 2015).

#### 3.1.1. Publications and citation trends

**Figure 3:**
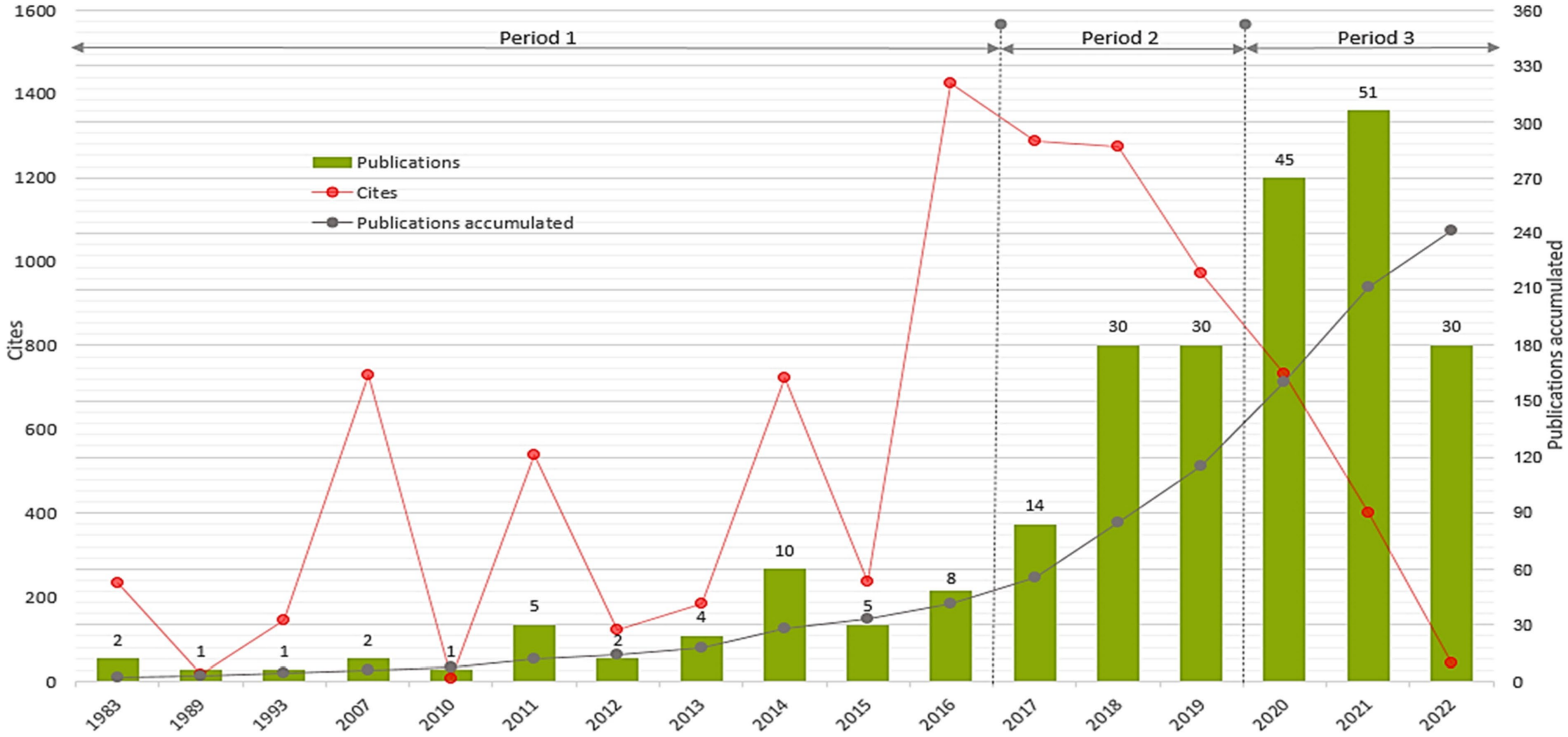
Distribution of publications and citations by year (1983–2022).

With bibliometric analysis of 241 articles included in the analysis, we determined the distribution by year of publication, accumulated number of publications, and the average citations per article (López-Robles et al., 2021). We displayed the graphical representation of the results in Figure 3.

As can be seen in Figure 3, most of the articles were published during 2020 and 2021, and a downward trend is observed during 2022. The growth in number of studies addressing digital addiction and depression during 2020–2021 could result from the global prevalence of COVID-19 during these years. COVID-19 pandemic had significant implications not only for physical health but also for psychological well-being due to both health hazards and also macroprudential policies such as lockdowns. As a result, people were more exposed to the use of digital technologies for not only work or schooling but also for socializing and coping with the threats of pandemic.

#### 3.1.2. Most influential authors

Within the scope of the 241 articles analyzed, a total of 995 authors investigated the relationship between digital addiction and depression. Some of these authors have been involved in more than one articles. We determined the top 10 most productive authors that contributed to the digital addiction-depression knowledge base according to the highest number of publications and associated citations. These authors are listed in Table 2 according to the number of citations their articles received.

**Table 2:** Top 10 authors most cited in the digital addiction–depression research field.

Rank	Author	TC*	TP	h-index
1	Elhai, Jon D	1,443	14	55
2	Hall, Brian J.	1,225	6	39
3	Levine, Jason C.	1,181	5	17
4	Dvorak, Robert D.	990	3	26
5	Yen, Cheng-Fang	744	8	42
6	Ko, Chih-Hung	672	6	49
7	Shensa, Ariel	651	6	25
8	Primack, Brian	649	5	42
9	Sidani, Jaime	649	5	23
10	Yen, Ju-Yu	642	5	35

*TC, total citations; TP, total publications. Data retrieved from WoS on August 27, 2022.

As shown in Table 2, Elhai made the greatest contribution to the field with a total of 14 publications cited in 1443 articles. Although most authors listed in Table 2 published closer number of articles, Hall and Levine made greater impact with a total of 1,225 and 1,181 citations, respectively. Similarly, Dvorak made a significant impact receiving 990 citations for only three articles he published so far.

#### 3.1.3. Most influential journals

We determined that articles addressing the digital addiction-depression relationship were published in 101 different journals between 1983 and 2022. The top 10 journals that published the highest number of articles are listed in Table 3, based on the total number of citations received.

**Table 3:** Top 10 journals most cited in the digital addiction–depression domain.

Rank	Journal Name	TC*	TP	JIF	JCI	Category quartile
1	*Computers in Human Behavior*	1,315	13	8.957	2.59	Q1
2	*Journal of Affective Disorders*	987	14	6.533	1.37	Q1
3	*Comprehensive Psychiatry*	482	8	7.211	1.31	Q1
4	*Psychiatry Research*	358	9	11.225	1.46	Q1
5	*Cyberpsychology Behavior and Social Networking*	335	10	6.135	1.31	Q1
6	*International Journal of Environmental Research and Public Health*	239	18	4.614	0.93	Q1
7	*Addictive Behaviors*	234	7	4.591	1.27	Q2
8	*Frontiers in Psychiatry*	104	8	5.435	0.94	Q2
9	*Frontiers in Psychology*	52	6	4.232	1.03	Q1
10	*Current Psychology*	17	6	2.387	0.73	Q2

*TC, total citations; TP, total publications; JIF, Journal Impact Factor™; JCI, Journal Citation Indicator.

Data retrieved from WoS on August 27, 2022.

As can be seen in Table 3, Computers in Human Behavior was the leading journal receiving a total of 1,315 citations for the 13 articles published. Journal of Affective Disorders (TC = 987), and Comprehensive Psychiatry (TC = 482) also contributed greatly to the field as the second and third leading journals in the digital addiction-depression research field. It is also noteworthy that journals with a psychology or psychiatry focus were more interested in publishing research on digital addiction-depression relationship, or researchers were more inclined to publish their research in these journals. International Journal of Environmental Research and Public Health Journal contributed the field with the highest number of articles, which is not surprising considering the fact that both digital addiction and depression have public health hazards.

#### 3.1.4. Most cited articles

The top 10 articles that received the highest number of citations among the 241 articles included in the analysis are listed in Table 4.

**Table 4:** Top 10 articles cited in the digital addiction–depression domain.

Rank	Article name	Journal name	Author(s)	Year	TC*
1	Problematic smartphone use: A conceptual overview and systematic review of relations with anxiety and depression psychopathology	*Journal of Affective Disorders*	Elhai, Jon D.; Dvorak, Robert D.; Levine, Jason C.; Hall, Brian J.	2017	527
2	The comorbid psychiatric symptoms of Internet addiction: Attention deficit and hyperactivity disorder (ADHD), depression, social phobia, and hostility	*Journal of Adolescent Health*	Yen, Ju-Yu; Ko, Chih-Hung; Yen, Cheng-Fang; Wu, Hsiu-Yueh; Yang, Ming-Jen	2007	467
3	#Sleepyteens: Social media use in adolescence is associated with poor sleep quality, anxiety, depression and low self-esteem	*Journal of Adolescence*	Woods, Heather Cleland; Scott, Holly	2016	439
4	Association Between Social Media Use and Depression Among Us Young Adults	*Depression and Anxiety*	Lin, Liu Yi; Sidani, Jaime E.; Shensa, Ariel; Radovic, Ana; Miller, Elizabeth; Colditz, Jason B.; Hoffman, Beth L.; Giles, Leila M.; Primack, Brian A.	2016	359
5	Fear of missing out, need for touch, anxiety and depression are related to problematic smartphone use	*Computers in Human Behavior*	Elhai, Jon D.; Levine, Jason C.; Dvorak, Robert D.; Hall, Brian J.	2016	330
6	Social network determinants of depression	*Molecular Psychiatry*	Rosenquist, J. N.; Fowler, J. H.; Christakis, N. A.	2011	284
7	Depression and Internet addiction in adolescents	*Psychopathology*	Ha, Jee Hyun; Kim, Su Yeon; Bae, Soojeong C.; Bae, Sujin; Kim, Hyungjun; Sim, Minyoung; Lyoo, In Kyoon; Cho, Soo Churl	2007	261
8	No More Fomo: Limiting Social Media Decreases Loneliness and Depression	*Journal of Social and Clinical Psychology*	Hunt, Melissa G.; Marx, Rachel; Lipson, Courtney; Young, Jordyn	2018	212
9	Use of multiple social media platforms and symptoms of depression and anxiety: A nationally-representative study among US young adults	*Computers in Human Behavior*	Primack, Brian A.; Shensa, Ariel; Escobar-Viera, Cesar G.; Barrett, Erica L.; Sidani, Jaime E.; Colditz, Jason B.; James, A. Everette	2017	180
10	Is video gaming, or video game addiction, associated with depression, academic achievement, heavy episodic drinking, or conduct problems?	*Journal of Behavioral Addictions*	Brunborg, Geir Scott; Mentzoni, Rune Aune; Froyland, Lars Roar	2014	170

*Data retrieved from WoS on August 27, 2022.

As presented in Table 4, the most influential article published within digital addiction-depression knowledge domain was written by the most influential authors demonstrated earlier (see Table 2). The most influential article was a conceptual paper addressing the relations between smartphone use and depression. Most of the articles had a focus on social media use-depression relationship (*n* = 6), followed by smartphone addiction (*n* = 2), internet addiction (*n* = 1), and video game addiction (*n* = 1).

#### 3.1.5. Most productive countries

We determined that the relationship between digital addiction and depression was studies in 43 different countries. The 10 most productive countries that contributed to the digital addiction-depression knowledge domain are listed in Table 5.

**Table 5:** Top 10 countries with most publications in the DA–depression domain.

Rank	Country	TP*	TC
1	China	76	2,888
2	The USA	70	3,616
3	South Korea	24	692
4	Turkey	22	639
5	Taiwan	14	991
6	Germany	12	181
7	England	11	374
8	Australia	10	258
9	Malaysia	6	194
10	Spain	6	369

*Data retrieved from WoS on August 27, 2022.

As can be seen in Table 5, China was the leading country with 76 studies published, and was followed by the United States with a closer number of articles published in this knowledge domain (*n* = 70). South Korea and Turkey also contributed to the field with 24 and 22 articles, respectively. It is noteworthy that both Eastern and Western countries were included in the list of most productive countries, which might imply the global research interest in the relationship between digital addiction and depression.

### 3.2. Science mapping and performance analysis

In this section, we report on the results of the science mapping analysis performed using SciMAT: (i) scientific evolution structure identified through period-based analysis, (ii) overlapping map, and (iii) thematic evolution structure.

#### 3.2.1. Scientific evolution structure

##### 3.2.1.1. Period 1 (1983–2016)

**Figure 4:**
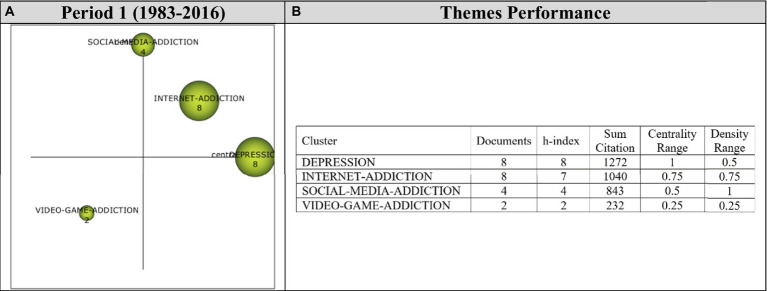
**(A)** Strategic diagram and **(B)** performance analysis.

Analysis of 41 articles published during the first period (1983–2016) revealed four main themes. Performance values and strategic diagram related to these themes are presented in Figure 4.

During Period 1, *Depression, Internet-Addiction* and *Social-Media-Addiction* themes emerged as motor themes. These are the main themes that contributed the most to the development of the field. The *Video-Game-Addiction* theme was found to be an emerging/declining theme. The themes of highest significance during this period were *Depression* and *Internet-Addiction* themes, represented by eight documents.

**Figure 5:**
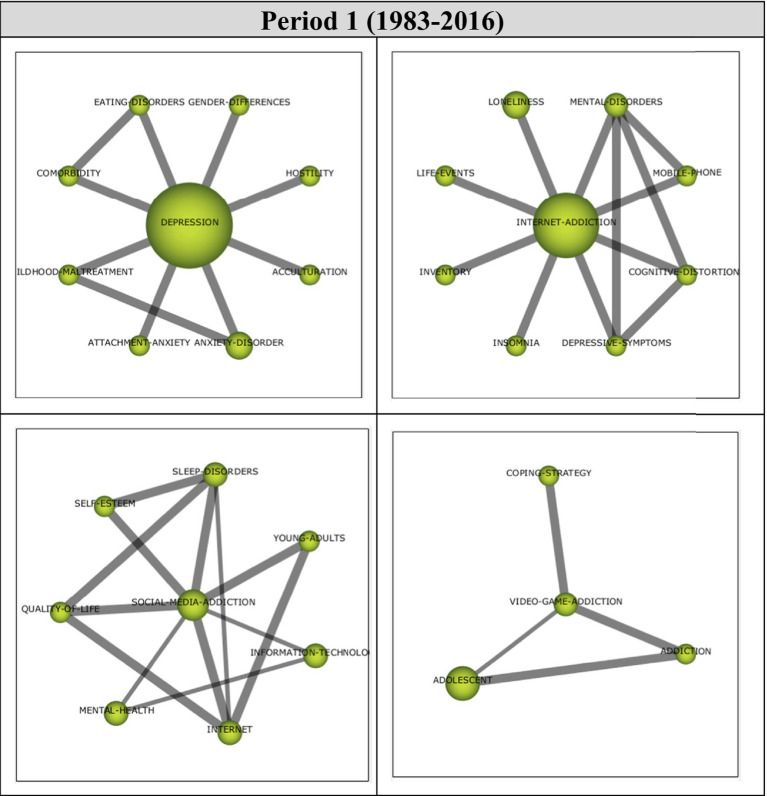
Thematic network structures.

Cluster networks (Figure 5) were examined in order to determine the topics related to the themes emerged during the first period (1983–2016). It was found that the main theme of *Depression* (1, 0.5) was strongly related to *Gender-Differences, Hostility, Acculturation, Anxiety-Disorder, Attachment-Anxiety, Childhood-Maltreatment, Comorbidity* and *Eating-Disorders* themes. Strong relations were evident between the main theme and the sub-themes. These studies on Gender-Differences (Liang et al., 2016), Hostility (Yen et al., 2011), Acculturation (Kim et al., 2012), Anxiety-Disorder (Hinic et al., 2010), Childhood-Maltreatment (Yang et al., 2014), Comorbidity (Cheung and Wong, 2011), and Eating-Disorders (Tao, 2013) support the sub-themes identified in the *Depression* cluster network.

The analysis revealed that the main theme of *Internet-Addiction* (0.75, 0.75) had strong relationships with *Mental-Disorders, Mobile-Phone, Cognitive-Distortions, Depressive-Symptoms, Insomnia, Inventory, Life-Events* and *Loneliness* themes. These studies on Mental-Disorders (Hinic et al., 2010), Mobile-Phone (Elhai et al., 2016), Depressive-Symptoms (Okamoto et al., 2011), Insomnia (Cheung and Wong, 2011), Inventory (Ha et al., 2007), Life-Events (Yang et al., 2014) and Loneliness (Özdemir et al., 2014) illustrate the sub-themes in the *Internet-Addiction* cluster network.

The main theme of *Social-Media-Addiction* (0.5, 1) was found to have strong relationships with *Sleep-Disorders, Young-Adults, Information-Technology, Internet, Mental-Health, Quality-Of-Life* and *Self-Esteem* themes. For example, these studies on Young-Adults (Lin et al., 2016), Information-Technology (Elhai et al., 2016), Internet (Lin et al., 2016), Mental-Health (Loton et al., 2016), Quality-of-Life (Afsar, 2013) and Self-Esteem (Woods and Scott, 2016) support the sub-themes identified in relation to the *Social-Media-Addiction* theme.

##### 3.2.1.2. Period 2 (2017–2019)

**Figure 6:**
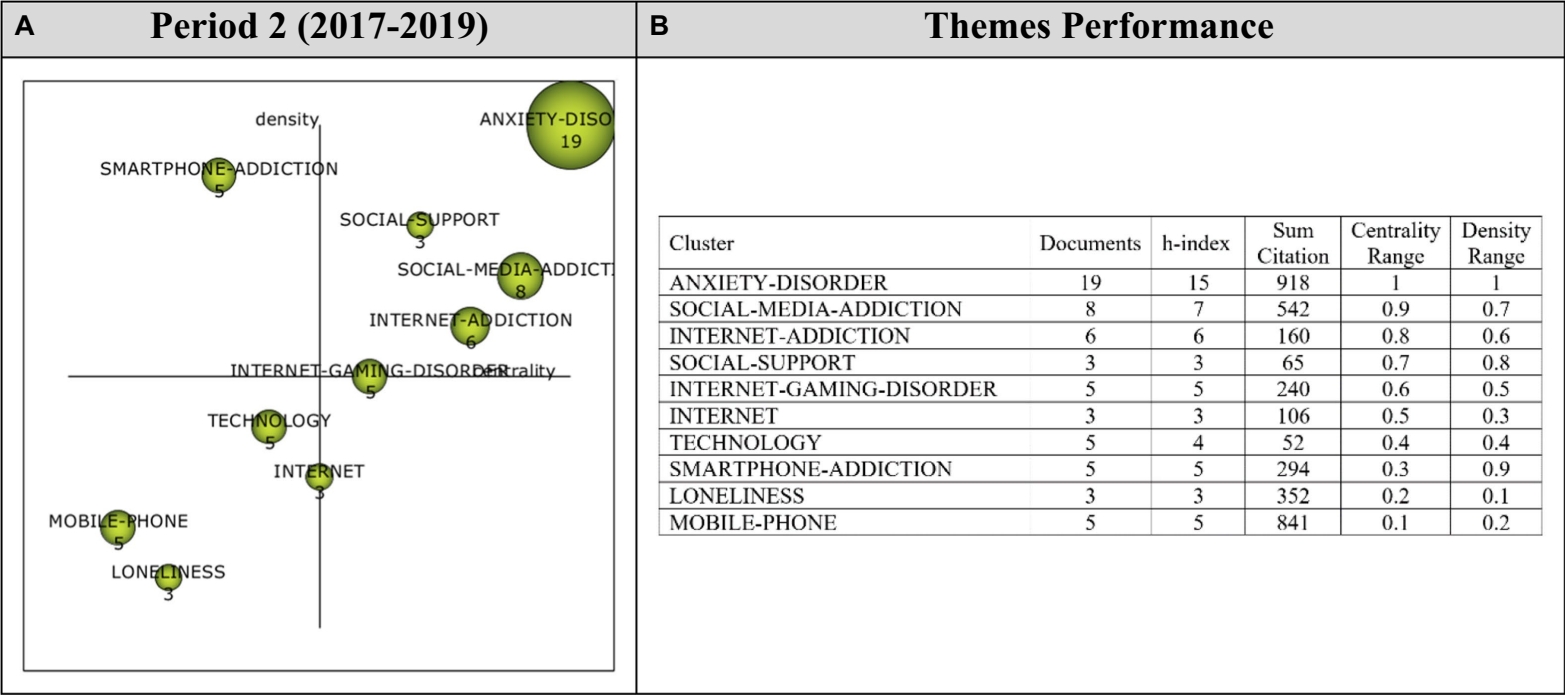
**(A)** Strategic diagram and **(B)** performance analysis.

Analysis of 74 articles published during the second period (2017–2019) revealed a total of ten main themes. Performance values and strategic diagram related to these themes are presented in Figure 6.

The theme with the highest importance among the themes that emerged during the second period (2017–2019) was the *Anxiety-Disorder* theme, represented by 19 documents. In addition, *Social-Media-Addiction, Internet-Addiction, Social-Support,* and *Internet-Gaming-Disorder* themes emerged as motor themes that contributed the most to the development of the field during this period. *Smartphone-Addiction* theme emerged as highly-specialized and isolated theme, which implies that *Smartphone-Addiction* theme had strong inter-relationships, but lacked the appropriate background or significance for the digital addiction-depression research field. *Mobile-Phone, Technology* and *Loneliness* themes were among the emerging/declining themes. The *Internet* theme was found to be a basic and transversal theme, which indicates that it was not developed in the digital addiction-depression knowledge domain despite being related to this research field.

**Figure 7:**
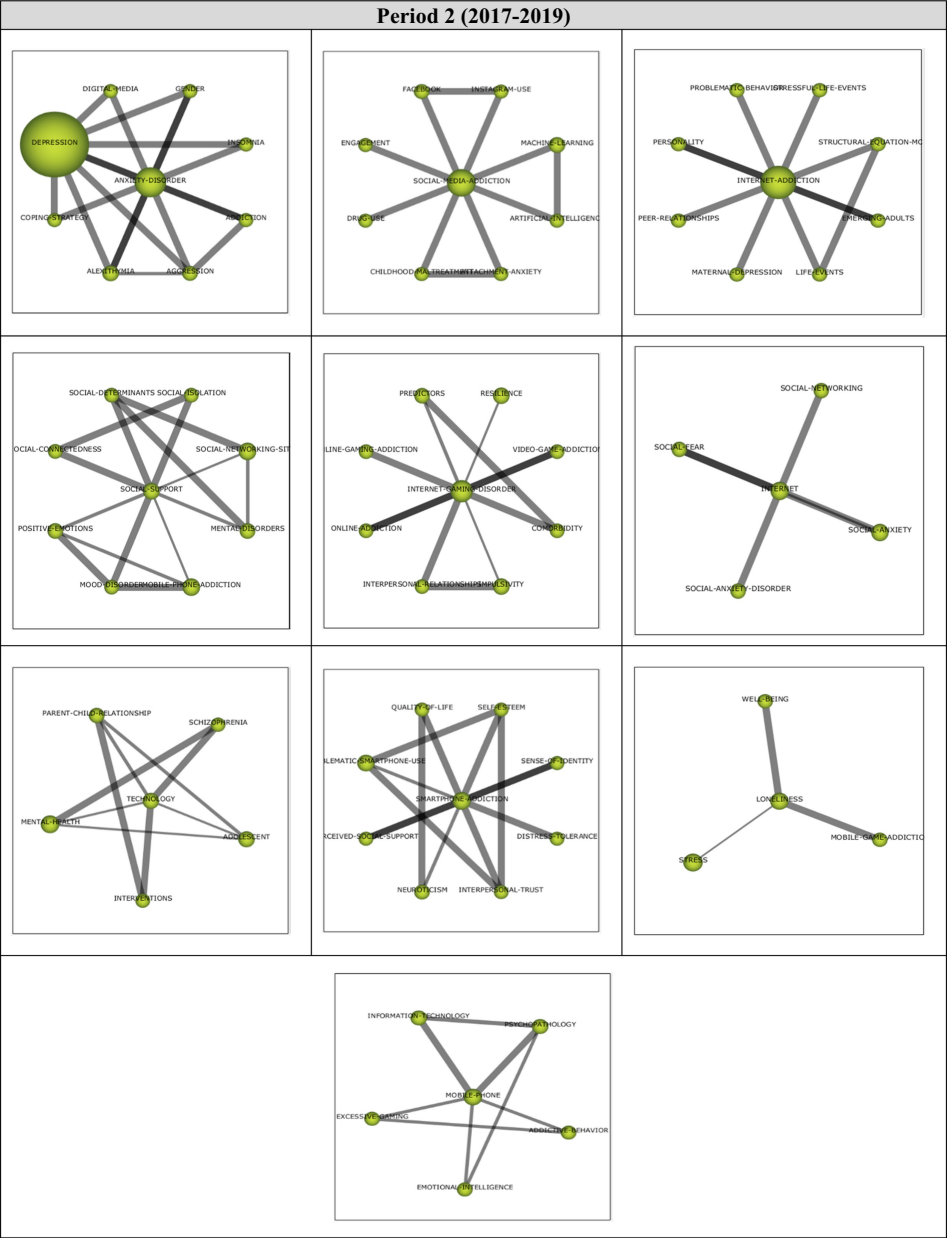
Thematic network structures.

Cluster networks of motor themes (Figure 7) were examined in order to determine the topics related to these motor themes guided research during the second period (2017–2019). Accordingly, the main theme of *Anxiety-Disorder* (1, 1) was found to have relationship with *Gender, Insomnia, Addiction, Aggression, Alexithymia, Coping-Strategy, Depression* and *Digital-Media* themes. To illustrate these sub-themes in the *Anxiety-Disorder* cluster network, these studies on Gender (Mitchell and Hussain, 2018), Insomnia (Evren et al., 2019), Addiction (Elhai et al., 2017a,b), Aggression (Obeid et al., 2019), Alexithymia (Gao et al., 2018), Coping Strategy (Chou et al., 2018), and Depression (Elhai et al., 2017a,b) could be cited.

The main theme of *Social-Media-Addiction* (0.9, 0.7) was determined to have a strong relationship with *Instagram-Use, Machine-Learning, Artificial-Intelligence, Attachment-Anxiety, Childhood-Maltreatment, Drug-Use, Engagement* and *Facebook* themes. These studies on Instagram-Use (Shensa et al., 2018), Machine-Learning (Cacheda et al., 2019), Artificial-Intelligence (Cacheda et al., 2019), Attachment-Anxiety (Worsley et al., 2018), Childhood-Maltreatment (Worsley et al., 2018), Drug-Use (Zhao et al., 2017), Engagement (Windler et al., 2019), and Facebook (Hunt et al., 2018) could be listed to exemplify the sub-themes in the *Social-Media-Addiction* cluster network.

The main theme of *Internet-Addiction* (0.8, 0.6) was found to have a strong relationship with *Stressful-Life-Events, Structural-Equation-Model, Emerging-Adults, Life-Events, Maternal-Depression, Peer-Relationships, Personality* and *Problematic-Behavior* themes. These studies on Stressful-Life-Events (Zhao et al., 2017), Structural-Equation-Model (Mak et al., 2018), Emerging-Adults (Przepiorka et al., 2019), Life-Events (Zhao et al., 2017) Maternal-Depression (Choi et al., 2018), Peer-Relationships (Zhou et al., 2017), Personality (Przepiorka et al., 2019), and Problematic-Behavior (Hsieh et al., 2018) can be to support the sub-themes in the *Internet-Addiction* cluster network.

The main theme of *Social-Support* (0.7, 0.8) was determined to have a strong relationship with *Social-Isolation, Social-Networking-Sites, Mental-Disorders, Mobile-Phone-Addiction, Mood-Disorder, Positive-Emotions, Social-Connectedness* and *Social-Determinants* themes. These studies on Social-Isolation (Domènech-Abella et al., 2019), Social-Networking-Sites (Hunt et al., 2018), Mental-Disorders (Elhai et al., 2017a,b), Mobile-Phone-Addiction (Yang et al., 2019), Mood-Disorder (Li et al., 2017), Positive-Emotions (Li et al., 2017), Social-Connectedness (Levula et al., 2018), and Social-Determinants (Puigpinós-Riera et al., 2018) exemplify the sub-themes in the *Social-Support* cluster network.

The main theme of *Internet-Gaming-Disorder* (0.6, 0.5) was found to have a strong relationship with *Resilience, Video-Game-Addiction, Comorbidity, Impulsivity, Interpersonal-Relationships, Online-Addiction, Online-Gaming-Addiction*, and *Predictors* themes. These sub-themes in the *Internet-Gaming-Disorder* cluster network can be illustrated with these studies on Resilience (Zhou et al., 2017), Comorbidity (Liu et al., 2018), Impulsivity (Obeid et al., 2019), Interpersonal-Relationships (Ryu et al., 2018), Online-Addiction (Burleigh et al., 2018), and Predictors (Mitchell and Hussain, 2018).

##### 3.2.1.3. Period 3 (2020–2022)

**Figure 8:**
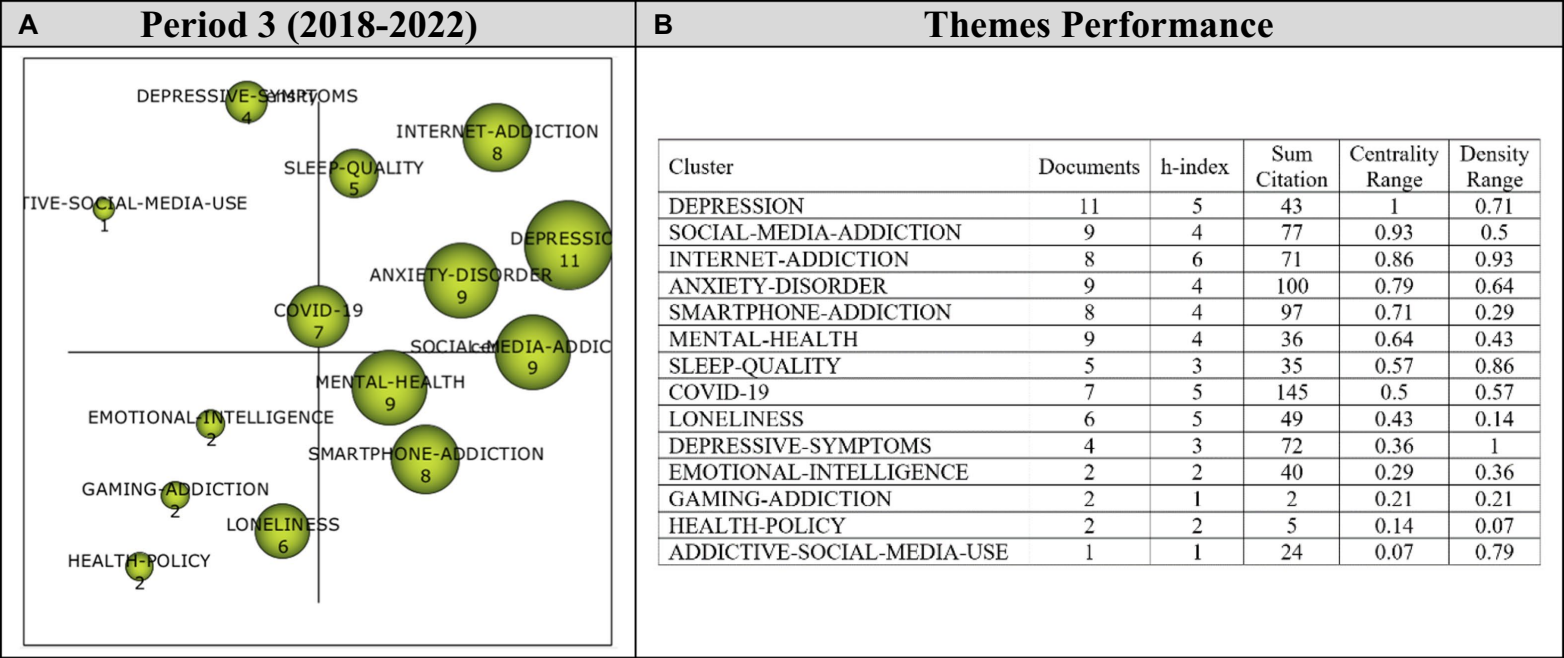
**(A)** Strategic diagram and **(B)** performance analysis.

Analysis of 126 articles published during the first period (2020–2022) revealed 14 main themes. Performance values and strategic diagram related to these themes are presented in Figure 8.

During the third period covering the years 2020–2022, the *Depression* theme was found to be the most significant theme, represented by 11 documents. During this period, it was observed that themes were spread to all four quartiles. While *Depression, Social-Media-Addiction, Internet-Addiction, Anxiety-Disorder, Sleep-Quality,* and *COVID-19* themes emerged as motor themes, *Depressive-Symptoms* and *Addictive-Social-Media-Use* themes were included in highly-developed and isolated themes. *Emotional-Intelligent, Gaming-Addiction, Loneliness,* and *Health-Policy* were, on the other hand, among the emerging/declining themes. *Mental-Health* and *Smartphone-Addiction* themes were found to be basic and transversal themes during the last period.

**Figure 9:**
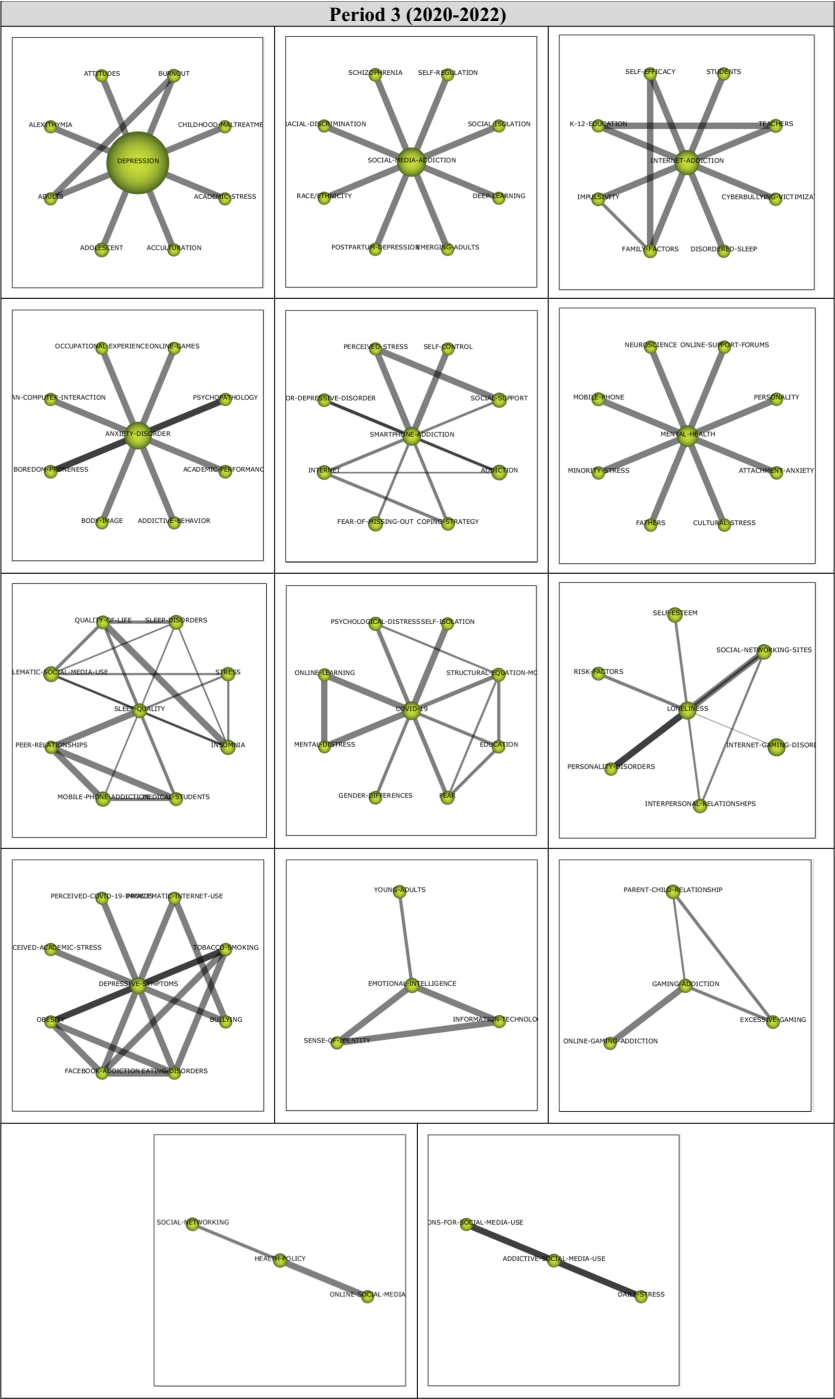
Thematic network structures.

Cluster networks of motor themes (Figure 9) were examined in order to determine the topics related to these motor themes during the last period (2020–2022). Results of the analysis showed that the main theme of *Depression* (1, 0.71) were strongly related to *Burnout, Childhood-Maltreatment, Academic-Stress, Acculturation, Adolescent, Adults, Alexithymia* and *Attitudes* themes. These studies on Burnout (Karakose et al., 2022c), Childhood-Maltreatment (Zeng et al., 2021), Academic-Stress (Zhang et al., 2022), Acculturation (Franco and Carrier, 2020), Adolescent (Tian et al., 2021), Adults (Elhai et al., 2020), Alexithymia (Lv et al., 2022), and Attitudes (Yu et al., 2021) illustrate the sub-themes in the *Depression* cluster network.

The main theme of *Social-Media-Addiction* (0.93, 0.5) was determined to have a strong relationship with *Self-Regulation, Social-Isolation, Deep-Learning, Emerging-Adults, Postpartum-Depression, Race/Ethnicity, Racial-Discrimination*, and *Schizophrenia* themes. These studies on Self-Regulation (Akıl et al., 2022), Social-Isolation (Sewall et al., 2022), Deep-Learning (Kim et al., 2022), Emerging-Adults (Cano et al., 2021), Postpartum-Depression (Shatte et al., 2020), Race/Ethnicity (Nereim et al., 2022), Racial-Discrimination (Pan et al., 2021), and Schizophrenia (Li et al., 2020) could be listed to support the emergent sub-themes in the *Social-Media-Addiction* cluster network.

The main theme of *Internet-Addiction* (0.86, 0.93) was found to be related to *Students, Teachers, Cyberbullying-Victimization, Disordered-Sleep, Family-Factors, Impulsivity, K-12-Education,* and *Self-Efficacy* themes. To illustrate these sub-themes in the *Internet-Addiction* cluster network, these studies on Students (Pereira et al., 2020), Teachers (Karakose et al., 2022c), Cyberbullying-Victimization (Wang et al., 2020), Disordered-Sleep (Masaeli and Farhadi, 2021), Impulsivity (Marzilli et al., 2020), K-12-Education (Karakose et al., 2022a), and Self-Efficacy (Chen et al., 2020) can be listed.

The main theme of *Anxiety-Disorder* (0.79, 0.64) was determined to have a relationship with *Online-Games, Psychopathology, Academic-Performance, Addictive-Behavior, Body-Image, Boredom-Proneness, Human-Computer-Interaction* and *Occupational-Experience*. These studies on Online-Games (Alhamoud et al., 2022), Psychopathology (Wolniewicz et al., 2020), Academic-Performance (Sayed et al., 2022), Addictive-Behavior (Kuru and Celenk, 2021), Body-Image (Hawes et al., 2020), Boredom-Proneness (Wolniewicz et al., 2020), Human-Computer-Interaction (Sewall et al., 2022), and Occupational-Experience (Zamora et al., 2021) exemplify the sub-themes in the *Anxiety-Disorder* cluster network.

The main theme of *Sleep-Quality* (0.57, 0.86) was found to be related to *Sleep-Disorders, Stress, Insomnia, Medical-Students, Mobile-Phone-Addiction, Peer-Relationships, Problematic-Social-Media-Use,* and *Quality-of-Life* themes. These studies on Sleep-Disorders (Jiang et al., 2022), Stress (She et al., 2021), Insomnia (Pohl et al., 2021), Medical-Students (Gong et al., 2021), Mobile-Phone-Addiction (Ivanova et al., 2020), Peer-Relationships (Feng et al., 2022), Problematic-Social-Media-Use (Majeed et al., 2020), and Quality-of-Life (Pohl et al., 2021) can be listed to illustrate the sub-themes in the *Sleep-Quality* cluster network.

The main theme of *COVID-19* (0.5, 0.57) was determined to have relationships with *Self-Isolation, Structural-Equation-Model, Education, Fear, Gender-Differences, Mental-Distress, Online-Learning,* and *Psychological-Distress* themes. To illustrate these sub-themes in the *COVID-19* cluster network, these studies on Self-Isolation (Júnior et al., 2021), Structural-Equation-Model (Elhai et al., 2020), Education (Elhai et al., 2020), Fear (Wolniewicz et al., 2020), Gender-Differences (Hou et al., 2020), Mental-Distress (She et al., 2021), Online-Learning (She et al., 2021), and Psychological-Distress (Karakose et al., 2022c) can be cited.

#### 3.2.2. Overlapping map

**Figure 10:**
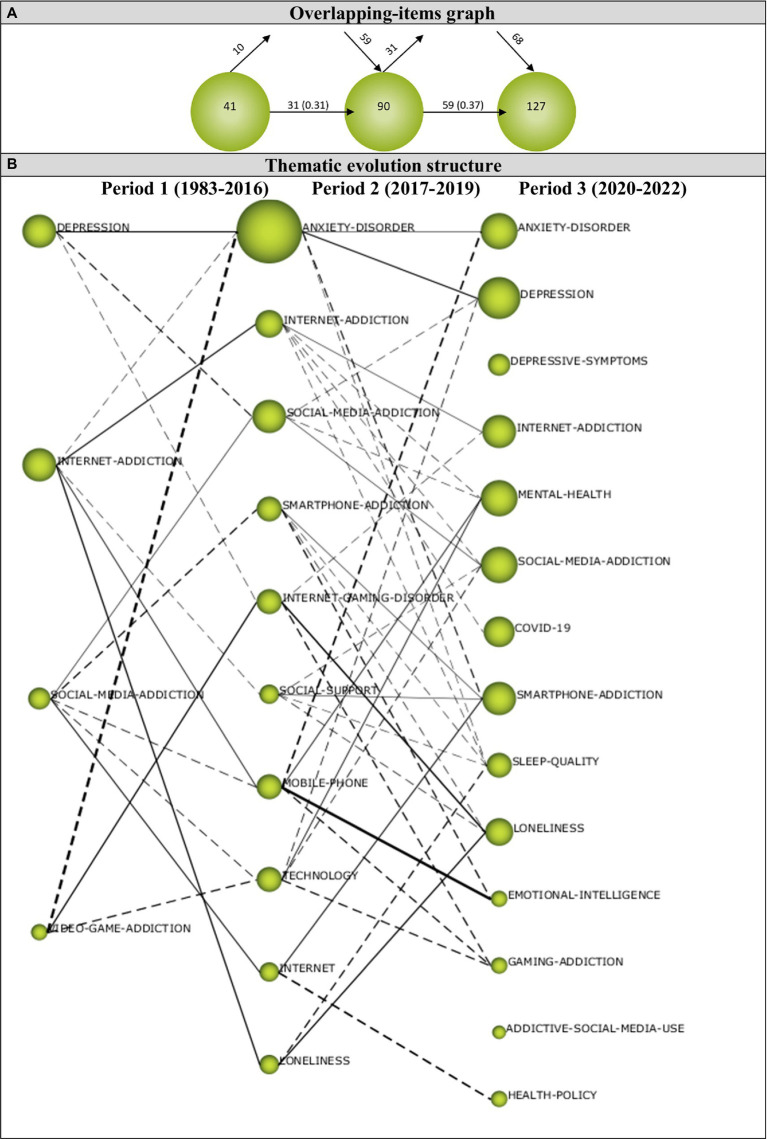
**(A)** Overlapping map and **(B)** thematic evolution map.

The overlapping-items graph shows the number of keywords in each period, identifies keyword that are newly appeared, lost, and reused in the consecutive period (Salazar-Concha et al., 2021). The overlapping map in Figure 10A shows that a total of 41 keywords were used during the first period, and among them 10 keywords were not used in the following period while 31 of them continued to be used. During the second period, 90 keywords were used in total, and 59 of these keywords were also used in the last period while 31 keywords were ceased to be used. During the third period, a total of 127 keywords were used. While the number of new keywords used for the first time during the second period was 59, it was calculated as 68 for the third period. However, it was determined that the similarity index between the sub-periods increased from 0.31 to 0.37, which indicates that the keywords were mostly shared between the periods. The overlapping-items graph revealed that the terminology related to the digital addiction and depression is getting stronger and stronger each year, and new terms have been introduced into this research field across the periods of analysis. The number of keywords used increased from 41 during the first period to 127 during the last period, which shows that research topics addressed with regard to the relationship between digital addiction and depression have diversified and increased cumulatively. The increase in the number of keywords added during each period shows that the studies on digital addiction and depression are constantly developing, and the disappearance of keywords shows that the keywords in this research field are constantly updated.

#### 3.2.3. Thematic evolution structure

The thematic evolution map (see Figure 10B) illustrates the pattern of development in knowledge domains over the analyzed periods, and the relationship between research topics regarding the digital addiction and depression relationship. The size of the spheres on the map shows the number of publications, and the thickness of the lines connecting the spheres shows the strength of the correlation between the themes emerged during the periods (Cobo et al., 2012; Murgado-Armenteros et al., 2015).

The thematic evolution map in Figure 10B shows that four themes emerged during the first period (1983–2016) which constitutes 17.01% (41 documents) of the articles. Among these themes, INTERNET-ADDICTION and SOCIAL-MEDIA-ADDICTION themes continued to exist in other periods, while DEPRESSION and VIDEO-GAME-ADDICTION themes were connected with different themes. The DEPRESSION theme transformed into ANXIETY-DISORDER, SOCIAL-MEDIA-ADDICTION, and INTERNET-GAMING-DISORDER themes during the second period. The INTERNET-ADDICTION theme formed relationships with the ANXIETY-DISORDER, SOCIAL-SUPPORT, MOBILE-PHONE, and LONELINESS themes during the second period. The SOCIAL-MEDIA-ADDICTION theme was linked to the SMARTPHONE-ADDICTION, MOBILE-PHONE, TECHNOLOGY, and INTERNET themes during the second period. VIDEO-GAME-ADDICTION theme changed into ANXIETY-DISORDER, INTERNET-GAMING-DISORDER and TECHNOLOGY themes during the second period. It was found that the highest h-index during the first period belonged to the DEPRESSION theme.

Ten themes emerged during the second period (2017–2019), constituting 30.71% (74 documents) of the articles analyzed. Two of these themes were transferred from the first period while eight of them appeared for the first time during the second period. Five of these themes from the second period continued to exist during the third period. All of the themes addressed during the second period were connected to the themes from the first and third period. ANXIETY-DISORDER, INTERNET-ADDICTION, SOCIAL-MEDIA-ADDICTION, SMARTPHONE-ADDICTION, and LONELINESS themes continued to exist during the last period. ANXIETY-DISORDER theme was linked to DEPRESSION, SMARTPHONE-ADDICTION, and SLEEP-QUALITY themes during the last period. The INTERNET-ADDICTION theme was connected to MENTAL-HEALTH, SOCIAL-MEDIA-ADDICTION, COVID-19, and SLEEP-QUALITY themes. The SOCIAL-MEDIA-ADDICTION theme was linked to DEPRESSION and MENTAL-HEALTH themes during the last period. The SMARTPHONE-ADDICTION theme was linked to SLEEP-QUALITY, LONELINESS, and EMOTIONAL-INTELLIGENCE themes during the last period while the INTERNET-GAMING-DISORDER theme was linked to INTERNET-ADDICTION, LONELINESS, and GAME-ADDICTION themes. The SOCIAL-SUPPORT theme was connected to SOCIAL-MEDIA-ADDICTION, SMARTPHONE-ADDICTION, SLEEP-QUALITY, and LONELINESS themes during the last period. The MOBILE-PHONE theme was transformed into ANXIETY-DISORDER, EMOTIONAL-INTELLIGENT, and GAMING-ADDICTION themes during the last period while the TECHNOLOGY theme was transferred into DEPRESSION, MENTAL-HEALTH, SOCIAL-MEDIA-ADDICTION, and GAMING-ADDICTION themes. The INTERNET theme changed into SMARTPHONE-ADDICTION and HEALTH-POLICY themes during the last period while the LONELINESS theme was connected to the SLEEP-QUALITY theme. It was found that the highest h-index during the second period belonged to the ANXIETY-DISORDER theme.

The third period (2020–2022) represented 52.28% (126 documents) of the articles analyzed, and 14 themes emerged during this period. The ANXIETY-DISORDER, INTERNET-ADDICTION, SOCIAL-MEDIA-ADDICTION, SMARTPHONE-ADDICTION, and LONELINESS themes continued to exist during this period from but the themes of DEPRESSION, DEPRESSIVE-SYMPTOMS, MENTAL-HEALTH, COVID-19, SLEEP-QUALITY, EMOTIONAL-INTELLIGENCE, GAMING-ADDICTION, ADDICTIVE-SOCIAL-MEDIA-USE, and HEALTH-POLICY appeared for the first time during the third period. DEPRESSIVE-SYMPTOMS and ADDICTIVE-SOCIAL-MEDIA-USE themes were not connected to any themes from the previous period. It was found that the highest h-index during the last period belonged to the INTERNET-ADDICTION theme.

## 4. Discussion

The current study investigated the intellectual and conceptual evolution of research addressing the digital addiction and depression relationship, and identified the changing research trends and underlying research themes in this knowledge domain through period-based science mapping analysis. The study also reflected on the leading scholars, journals, articles, and countries with regard to their contribution to the development of the field. Thus, the study yielded significant results that would guide future research in the field, and help develop its theory. In this section, we elaborate research findings with a particular focus on the prominent and emerging themes as well as their evolution across periods of analysis.

As can be expected, the themes emerged from the analysis were two-fold: digital addiction-related and depression-related. With regard to the first one, internet addiction was the most significant and prevailing theme across all three periods. During the first period of analysis (1983–2016), studies on the relationship between internet addiction and depression were focused on both the causes and outcomes of internet addiction, particularly addressing cognitive distortions, insomnia, life events, and loneliness. Despite acknowledging that it is hard to identify whether these factors are the cause or the effect of internet addiction, scholars contend that these problems in an individual’s life mattered in the case of addiction and depression. Cognitive distortion, for instance, is described as errors of logic that results in unrealistic evaluations of situations, inaccurate perceptions of oneself and often manifests itself through selective abstract-focusing, overgeneralization, personalization, and polarized thinking (Beck et al., 1979). Thus, cognitive distortion was considered to have a significant role in both mental disorders such as major depression (Moss-Morris and Petrie, 1997; Henriques and Leitenberg, 2002) and in addictive disorders (Young, 2007; Decker and Gay, 2011; Celik and Odacı, 2013). In fact, Davis (2001) first proposed that cognitive distortions had an important role in internet addiction arguing that such errors of thought as rumination, negative self-appraisal or polarized thinking could result in problematic and extensive use of the internet. Davis’s (2001) hypothesis was later tested several times, and gained significant evidence (Young, 2015), which could also explain why cognitive distortions were focus of research during the first period.

Similarly, sleep problems such as insomnia was associated with increased risk of both internet addiction and depression (Cheung and Wong, 2011; Tan et al., 2016; Fang et al., 2019), in which sleeping problems could be the antecedent, consequence or the mediating factor (Chang et al., 2022). Young (2004) asserted that changes in one’s sleeping patterns could be the first warning sign of addiction, and the Diagnostic and Statistical Manual for Mental Disorders (DSM-5) (2013) lists sleep problems among the nine criteria of internet gaming disorder. Sleep problems are also among the prevalent symptoms of depression (World Health Organization (WHO), 2022). As a result, insomnia as a major sleep disturbance emerged among the significant themes of initial research addressing depression and internet addiction. In the same vein, loneliness was one of the central topics of research in both addiction and depression literature, and it was considered as a significant social factor having bidirectional relationships with both digital addiction (Dresp-Langley, 2020) and depression (Cacioppo et al., 2006; Erzen and Çikrikci, 2018). For instance, Cao et al. (2011) showed that young people with internet addiction tended to use the internet to cope with their loneliness. Likewise, Demir and Kutlu (2016) found that internet addiction mediated the relationship between loneliness and depression. Later research also added to this line of argument revealing that loneliness is also a significant factor in smartphone or social media addiction, and also in their association with depression (Hawi and Samaha, 2016; Hunt et al., 2018; Peper and Harvey, 2018; Devi and Dutta, 2022).

Another prominent theme during the first period of analysis was found to be “depression,” as can be expected. The cluster network showed that, as in the case of internet addiction, this research was mostly focused on identifying common factors underlying both addiction and depression as the sub-themes such as comorbidity, attachment anxiety, and acculturation suggested. Attachment anxiety refers to a person’s feelings of insecurity, anxiety, and fear in their relationship with others, which fundamentally results from an unhealthy relationship with primary caregivers during infancy (Bowlby, 1969; Ainsworth, 1989). The attachment theory proposes that people having attachment anxiety are likely to display maladjustment and interpersonal problems, and engage in compensatory responses to restore security (Li and Kato, 2006; Abeyta et al., 2015). Predicating on this perspective, attachment anxiety was also associated with dependency building and addiction, and as a recent review showed, the attachment theory has become one of the most frequently used theories in digital addiction research (Sun and Zhang, 2021). Attachment anxiety is also closely linked with depression (Bowlby, 1969) because people with attachment anxiety have difficulty regulating their emotions, managing their distress, and coping with negative life events (Mikulincer and Shaver, 2018; Zheng et al., 2020). This dual-relationship of attachment anxiety with depression and addiction could explain its emergence as a significant theme in research conducted during the first period of our analysis.

Another significant theme investigated in relation to depression was acculturation, a term used to define “the process whereby immigrants change their behavior and attitudes toward those of the host society” (Rogler et al., 1991, p. 585). As Hunt et al. (2004) states, acculturation is frequently addressed as a variable in health research predicating on the fact that cultural beliefs have implications for people’s health through determining their behavioral choices and attitudes. In addition, acculturative stress, which emanates from the challenging process of cultural adaptation (Ehlers et al., 2009), could influence people’s psychological well-being and result in disruptive behaviors like addiction, particularly due to loss of social support or increased family/social conflicts (Berry, 2003; Lorenzo-Blanco et al., 2012; Jin and Berge, 2015). Evidence provided for the potential negative behavioral and emotional outcomes of acculturation might have guided digital addiction-depression research.

Research during the first period of analysis did not only focus on internet addiction, but also addressed mobile phone addiction and social media addiction. In fact, during the first half of this first period, mobile phone was just a tool for communication, but within a decade, it turned out to be a necessity with all of its enthralling products and services (Peng et al., 2022). Therefore, mobile phone just emerged as a sub-theme of internet addiction because most of its products and services required internet connection. Social media addiction, on the other hand, emerged as a prevalent theme. This could be due to the fact that the fast-growth of interest in social networking sites, especially after the initiation of Facebook in 2004, might have been reflected in research particularly through the last decade of the first period. Closer scrutiny into the sub-themes of social media addiction showed that research addressing social media addiction and depression attempted to identify casual relationships between the two, with a particular focus on self-esteem, quality of life, and sleep disorders mostly in groups of young adults. Self-esteem, as defined by the social cognitive theory (Bandura, 1986), refers to people’s cognitive judgment of their ability to engage in a behavior, and thus determines how they will act. As a result, self-esteem was often shown to have a significant relationship with different types of addiction (Lin et al., 2008; Yu et al., 2016). Self-esteem also has psychological implications as higher levels of self-esteem is often associated with better psychological adjustment and positive affect while lower self-esteem could contribute to depression (Sowislo and Orth, 2013; Wang et al., 2018). Research also indicated that self-esteem could be directly related to forms of digital addiction (Błachnio and Przepiorka, 2016; Woods and Scott, 2016) or could moderate the relationship between digital addiction and depression (Zhang et al., 2015). With regard to the quality of life, Cheng and Li (2014) revealed that quality of real life, both on personal or societal level, correlated negatively with digital addiction, and argued that people could resort to the excessive use of the internet, or internet-related activities such as social media for that matter, in order to compensate for the perceived low quality of their life. Similarly, previous research showed reciprocal relations between excessive or pathological use of the social media and lower satisfaction with life (Chou and Edge, 2012; Kuss et al., 2014), as well as between depression and lower life satisfaction (Koivumaa-Honkanen et al., 2004; Swami et al., 2007). These results provided by early research seems to have guided research addressing social media addiction-depression relationship during this first period.

As for the second period of analysis comprising research published between 2017 and 2019, internet addiction and social media addiction maintained their significance as motor themes. Some of the sub-themes closely related to depression during the first period of analysis such as attachment anxiety (or childhood maltreatment) was more closely linked to social media addiction during the second period. On the other hand, research focusing on internet addiction continued to center on personal and social factors such as personality, peer relationships, and stressful life events. Yet, another sub-theme that emerged was structural equation modeling (SEM), which implied that most of the studies investigated the mediating/moderating effects in the relationship between addiction, depression and relevant personal/social factors. Another motor theme emerged during the second period was the internet gaming disorder, which was the only type of digital addiction included in Section III (the research appendix) of the fifth edition of the Diagnostic and Statistical Manual of Mental Disorders (DSM-5) as a possible condition requiring more evidence (American Psychiatric Association (APA), 2013; Moretta et al., 2022). Since then, growing research evidence showed that internet gaming disorder interacted with depression because it shared the same neural mechanisms as depression, and children or adolescents could be more vulnerable to this interaction due to their immature brain development (Liu et al., 2018; Jeong et al., 2019).

During the second period, depression did not emerge as a major theme, but was found to be a sub-theme of “anxiety disorder” theme, which also comprised other psychological problems such as insomnia, aggression, coping strategies, and alexithymia. This finding could imply that research during this period included wider variety of psychological symptoms that could underlie both depression and forms of digital addiction. Alexithymia seems to have attracted greater research interest during this period. In fact, alexithymia is not a new concept in the field of psychology research, but was first described by Sifneos (1973) as a constriction in emotional functioning, poverty of fantasy life, and inability to describe emotions. Later research correlated alexithymia with several factors including mental and substance-use disorders (Conrad et al., 2009; Morie et al., 2016), as well as pathological internet use (Lyvers et al., 2016) because people with alexithymia tended to be more impulsive, and more distressed (Lyvers et al., 2021). As a result, alexithymia was often linked with anxiety disorders and depression (Marchesi et al., 2000). The evidence provided by the previous psychological research seems to have guided scholars investigating the relationship between digital addiction and depression, especially during our second period of analysis.

Social support was found to be another significant theme that attracted growing research interest during the second period. As stated by Cohen (2004), social support provides people with psychological resources that promote their ability to cope with stress. Therefore, lower levels of social support could harm psychological well-being (Keles et al., 2020; Özbek and Karas, 2022) and could result in digital addiction (particularly smartphone or social media addiction) because people tend to seek social support on these platforms (Herrero et al., 2019). When all these variables investigated with regard to digital addiction-depression relationship considered as a whole, problematic coping mechanisms are arguably placed in juxtaposition with both addiction and depression. With this perspective, people’s inability to cope with real-life problems or psychological suffering could both decrease their well-being, and lead to digital addiction as a result of this faulty coping mechanisms (Wang et al., 2018; Marzilli et al., 2020).

During the last period of analysis between years 2020 and 2022, the same themes from the first and second period continued to garner research interest, but some recent concepts were also included as major or sub-themes. For instance, studies focusing on internet addiction and depression also addressed cyberbullying, victimization, and K-12 education. Cyberbullying is a new form of harassment defined as “the behavior of individuals or groups repeatedly sending hostile or offensive messages through electronic or digital media, with the intention of causing harm or discomfort to others while cybervictimization refers to being a victim of this behavior” (Wang et al., 2020, p. 2). The widespread use of the internet and digital technologies increased the risk for cybervictimization (Brochado et al., 2017; Liu et al., 2021). Research evidenced that cybervictimization highly correlates with digital addiction (Chang et al., 2015; Liu et al., 2021) and could elevate the risk of depression (Li et al., 2018). Like cyberbullying, the combined influence of digital addiction and depression on students’ academic achievement emerged as a rather recent phenomenon, and was found to be the sub-theme of anxiety disorder theme in the current analysis. Scholars argued that depression resulting from digital addiction could cause academic failure (Nam et al., 2018; Aydin et al., 2021; Foroughi et al., 2022), and academic failure could result in negative psychological states which could eventually lead students to seek comfort through online activities (Baturay and Toker, 2017). Smartphone addiction, which did not emerge as a prevalent theme during the previous periods, was found to be a basic and transversal theme during this last period. This finding indicates that smartphone addiction is a significant theme for the development of the digital addiction-depression research field, but warrants further investigation to be developed better.

### 4.1. Implications and limitations

The current study contributed to the literature through revealing the intellectual and conceptual architecture of research addressing the relationship between digital addiction and depression. The results suggest some implications for both theory and practice.

As the current results showed, research addressing digital addiction-depression relationship predicated on existing hypothesis and evidence in both addiction and depression literature, and as the thematic analysis revealed, scholars addressed many of the significant factors underlying depression and addiction. However, the current analysis also identified some gaps in this knowledge domain. First of all, research accumulated a significant knowledgebase regarding addiction-depression relationship in adolescents and young adults. Despite acknowledging that these groups are particularly vulnerable to the negative outcomes, emerging evidence also indicates that children (Sohn et al., 2019), adults (Aydin et al., 2021), and elderly are also risk groups (Özbek and Karas, 2022), perhaps for different underlying reasons. Therefore, we suggest that future studies could evaluate similar variables and their relationship in cohorts of children, adults, and elderly.

Similarly, the current analysis revealed that research focused on three types of digital addiction (i.e., internet addiction, social media addiction, internet gaming disorder), and closer scrutiny on their sub-themes showed that Facebook and Instagram were central in social media addiction research. In fact, digital addiction does not only refer to online activities, but also includes addiction to offline digital technologies such as video game addiction, sms addiction or addiction to mobile apps (with offline options; Basel et al., 2020; Karakose et al., 2022c). In the current analysis, we only observed the video-game addiction during the first period of analysis, and it was found to have lost research interest. Likewise, although Facebook and Instagram are popular sites, social media includes several other platforms such as YouTube, Snapchat, Twitter, Tumblr, or TikTok, or Reddit, which offer similar or distinct facilities of their own. For instance, a recent study stated that adolescents use YouTube (85%) more than Instagram (72%) and Facebook (51%), and a significant number of adolescents use Snapchat (69%), or Twitter (32%) (Vidal et al., 2020). As a result, future research is warranted to reveal the relationship of their problematic use with addiction and depression.

Social media addiction is rather a recent phenomenon in digital addiction literature, and it is considered to impact people negatively from several aspects. For example, there is public concern and research evidence on how social media could harm people’s cognitive evaluations of themselves or others, exhibits a false view of life, and damage sense of reality since content on social media platforms are built by their users, and might not reflect the reality (Cohen et al., 2018). As a result, as some studies showed, social media could lower people’s self-esteem and lead them to develop negative image of themselves, which have implications for their health and well-being (Saiphoo and Vahedi, 2019; Young et al., 2023). In the current study, body-image and eating disorders, for instance, emerged as the sub-themes of social media addiction during the last period of analysis. However, it is evident that much empirical evidence is necessary to develop deeper insights and to device necessary interventions for alleviating potential hazards (Fineberg et al., 2022).

Another implication for research is related to smartphone addiction theme. As mentioned earlier, smartphone addiction appeared as basic and transversal theme during only the last period of analysis (2020–2022), which implies that much research is necessary to develop this knowledgebase with regard to the relationship between smartphone addiction and depression. In fact, smartphones are perhaps the most integral type of digital technology in our lives, and they provide significant convenience in addition to their addictive potential. Furthermore, smartphone usage comprises the other potential sources of digital addiction since they are often used as a means of connecting to the internet or social media sites, and offer other facilities such as messaging or gaming. Therefore, understanding the patterns and outcomes of its problematic use is crucial, particularly with regard to mental health.

Although the current study presented significant results for the investigation of the relationship between digital addiction and depression, it also has some limitations. For one thing, the current study is different from traditional review studies that aim to present the cumulative results emanating from the synthesis of previous findings. Our study uses meta-data to reveal the bibliometric and conceptual features of digital addiction-depression knowledgebase, and highlights the sufficiently or under-investigated aspects as well as the evolutionary trends in its research. As a result, the current analysis does not reflect the findings of previous research. In addition, data for the current study was gathered from journal articles indexed in WoS. Although WoS covers a large scope of quality journals from various fields of study, and co-word analysis enables to reach broader scope of research, the current study may have missed some relevant research. Likewise, the data set does not include books, book chapters, conference proceedings, or dissertations. Therefore, the results of the present study should be interpreted within this scope.

## 5. Conclusion

Through combining bibliometric and science mapping analysis, this study delineated the conceptual and intellectual evolution of research that addressed the relationship between different types of digital addiction and depression. The results of science mapping analysis over three consecutive periods revealed that internet addiction was the most prominent theme studied in relation to depression during all three periods, and was followed by social media addiction and internet gaming disorder. It was also evident that researchers mostly addressed variables underlying both addiction and depression such as cognitive distortion, insomnia, life events, loneliness, self-esteem, attachment anxiety, acculturative stress, social support, and alexithymia. The study also showed that the relationship between smartphone addiction and depression garnered less research interest, so future studies could contribute greatly to the development of its knowledgebase.

Digital technologies have contributed substantially to human life through numerous benefits and conveniences they provided, but they have also brought with them potential hazards for the psychical and mental health of people. Existing research evidenced some of these negative outcomes, and raised significant concerns. Yet, there is always much to be uncovered in the face of the unprecedented scope of technological innovations in the modern age.

## Data availability statement

The raw data supporting the conclusions of this article will be made available by the authors, without undue reservation.

## Author contributions

TK: conception and design of study. TK and TT: acquisition of data. TK, BY, TT, and AK: analysis and/or interpretation of data, revising the manuscript critically for important intellectual content, and approval of the version of the manuscript to be published. TK, BY, and TT: drafting the manuscript. All authors contributed to the article and approved the submitted version.

## Conflict of interest

The authors declare that the research was conducted in the absence of any commercial or financial relationships that could be construed as a potential conflict of interest.

## Publisher’s note

All claims expressed in this article are solely those of the authors and do not necessarily represent those of their affiliated organizations, or those of the publisher, the editors and the reviewers. Any product that may be evaluated in this article, or claim that may be made by its manufacturer, is not guaranteed or endorsed by the publisher.
